# Bosutinib-Induced Pleural Effusion—Class Effect and Cross-Intolerance to All Tyrosine Kinase Inhibitors

**DOI:** 10.3390/hematolrep17010007

**Published:** 2025-01-31

**Authors:** Nikhil Vojjala, Hizqueel A. Sami, Nikhil Kumar Kotla, Supriya Peshin, Kanika Goyal, Soumya Kondaveety, Rishab Rajendra Prabhu, Geetha Krishnamoorthy

**Affiliations:** 1 Department of Internal Medicine, Trinity Health Oakland Hospital, Pontiac, MI 48341, USA; hizqueelahmed.sami@trinity-health.org (H.A.S.); nikhilkumar.kotla@trinity-health.org (N.K.K.); kanika.goyal@trinity-health.org (K.G.); rishabrajendra.prabhu@trinity-health.org (R.R.P.); geetha.krishnamoorthy@trinity-health.org (G.K.); 2 Department of Internal Medicine, Wayne State University School of Medicine, Detroit, MI 48201, USA; 3 Department of Internal Medicine, Norton Community Hospital, Norton, VA 24273, USA; supriya.peshin2@balladhealth.org; 4 Department of Hospital Medicine, Cleveland Clinic Mercy Hospital, Canton, OH 44708, USA; kondavs@ccf.org

**Keywords:** pleural effusion, bosutinib, tyrosine kinase inhibitors

## Abstract

**Introduction:** Tyrosine kinase inhibitors (TKIs) serve as the backbone in the management of chronic myelogenous leukemia and Philadelphia-positive Acute lymphoblastic Leukemia (Ph+ve ALL). With the growing use of TKIs, there has been an increase in adverse events related to these agents. Hereby, we present elderly women with Ph+ve ALL who developed recurrent pleural effusion, which was managed by switching the TKI and highlighting pleural effusion due to a third-generation TKI Bosutinib, adding to the minimal available literature. **Case Description:** Our patient is a 79-year-old female with Ph+ve ALL diagnosed in 2015 and started on treatment. She is also on TKI maintenance initially with Imatinib later shifted to second-generation TKIs. She started developing worsening dyspnea related to pulmonary toxicity related to TKI in the form of pleural effusion. Pleural effusion was initially managed with diuretics, later requiring thoracocentesis. Because of persistent pleural effusion, she was changed to multiple TKIs and finally started on Bosutinib. She even developed progressive pleural effusion while on Bosutinib which is managed by thoracocentesis. **Conclusions:** Through this case report, we would like to highlight refractory recurrent pleural effusion caused by bosutinib adding to the minimal available literature. In addition, we highlight the various treatment options in patients having cross-intolerance to various TKIs, especially pulmonary toxicity, and ponatinib might be a suitable option in such cases.

## 1. Introduction

Balanced translocation of the Breakpoint Cluster region (BCR) on chromosome 22 and the Abelson (ABL) gene on chromosome 9 creates a BCR-ABL fusion gene also called the Philadelphia chromosome. This fusion creates a constitutively activated tyrosine kinase, which is the signature genetic abnormality in patients with Chronic Myelogenous Leukemia (CML) and Philadelphia-positive Acute Lymphoblastic leukemia (Ph+ve ALL). In the early 2000s, it was demonstrated that inhibition of this fusion tyrosine kinase activity has a significant anti-leukemic effect, which was demonstrated in patients in a phase 1 dose-escalation study performed on patients with CML and, subsequently, Ph+ve ALL. Since then, these tyrosine kinase inhibitors (TKIs) have become the backbone and changed the management landscape of CML and Ph+ve ALL [1,2]. There has been significant progress in this area from the generic first-generation TKI Imatinib to the currently available multiple TKIs spanning across three generations. With the introduction of multiple therapeutic armamentaria, there has also been an increase in adverse events related to these agents. One among them is fluid retention and the third spacing [3]. Though it is a class effect, the propensity to cause this adverse event varies among various agents [4]. Hereby, we present elderly women with Ph+ve ALL who developed recurrent pleural effusion, which was managed by switching the TKI and highlighting pleural effusion due to a third-generation TKI Bosutinib, adding to the minimal available literature.

## 2. Case Presentation

**Figure 1:**
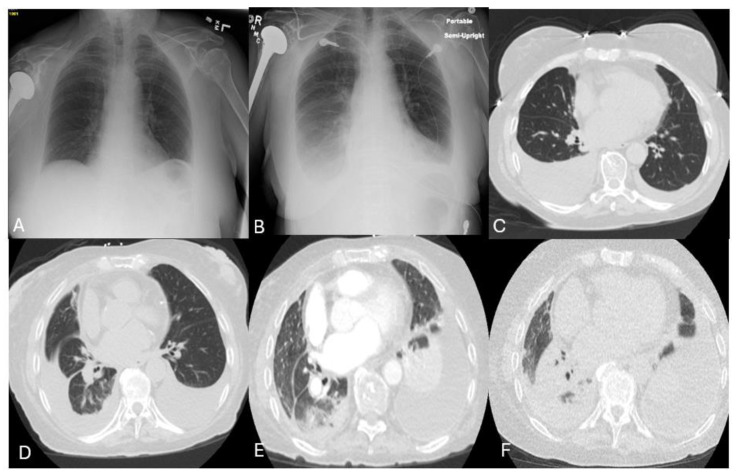
Radiological imaging of the index patient across various time frames. Radiological imaging of the index patient. (**A**) Baseline Chest X-ray before TKI initiation which is normal. (**B**) Chest X-ray performed in January 2018 while being on Dasatinib maintenance. (**C**) CAT scan performed in January 2018 while on Dasatinib, which shows bilateral pleural effusion (right-sided more than left-sided). (**D**) CAT scan performed while on Nilotinib therapy; this time pleural effusion is dominant on the left sided, while the right side is reduced. (**E**) CAT scan in 2022 while on Bosutinib. (**F**) CAT scan performed during index admission showing worsening left-sided pleural effusion with underlying lung collapse and right-sided pleural effusion with underlying collapse/consolidation in the form of air bronchograms.

Our patient is a 79-year-old woman with Philadelphia-positive Acute Lymphoblastic Leukemia (PH+ ALL), diagnosed in November 2015 when she presented with mouth sores and rectal bleeding. She was started on induction chemotherapy as per the GRAAPH-2005 protocol, and the tyrosine kinase inhibitor (TKI) Imatinib was added due to a positive Philadelphia chromosome. She developed worsening thrombocytopenia after about 2 months of the treatment. An extensive workup was performed and she was eventually diagnosed with immune thrombocytopenia, which was managed with intravenous immunoglobulin and Rituximab. Chemotherapy for ALL was transiently withheld until the platelet count recovered to a non-critical range. Subsequently, she was on Eltrombopag maintenance from 2017, which was later discontinued in 2021 once platelet counts improved. Imatinib-induced thrombocytopenia was also considered at that time; in addition, due to the persistent measurable residual disease (MRD), Imatinib was changed to second-generation TKI, Dasatinib. Subsequently, she started POMP (Prednisone + Vincristine + Methotrexate + Mercaptopurine) maintenance therapy along with Dasatinib, which she completed in November 2016. Remission induction phase 2 with a modified BFM (Berlin–Frankfurt–Munster) regimen was started in December 2016 and completed in June 2017. Later, she received 23 cycles of consolidation chemotherapy, which she completed in July 2019. Consolidation treatments were interrupted and complicated with intercurrent infections and cytopenia, which were managed accordingly (Figure 1).

In January 2018, while being on consolidation chemotherapy and Dasatinib maintenance, she developed worsening shortness of breath. A chest X-ray showed bilateral pleural effusion (Figure 1B). Subsequently, a Computerized Axial Tomography (CT) scan was performed, which showed bilateral pleural effusion predominantly on the right side (Figure 1C). Thoracentesis was performed, which showed exudative pleural effusion, but infective workup and cytology were negative. She continued to be in hematological remission from Ph + ALL. Subsequently, Dasatinib-induced pleural effusion was suspected, and the dose was reduced from 100 mg daily to 50 mg daily. Meanwhile, she was symptomatically managed with diuretics. After dose reduction, pleural effusion improved; however, she has persistent MRD warranting a dose increase to 100 mg daily. The same dose of 100 mg daily was continued while being on diuretics till March 2019. Given evidence of molecular relapse and persistent pleural effusion, Dasatinib was changed to a third-generation TKI, Nilotinib. So, she continued to take consolidation chemotherapy and Nilotinib maintenance till January 2022. She developed worsening shortness of breath from her baseline Modified Medical Research Council (MMRC) grade 1 while being on diuretics to MMRC grade 3. A repeat CT scan showed worsening pleural effusion this time on the left side with persistent right-sided mild effusion (Figure 1D). Repeat thoracentesis was performed, which revealed exudative effusion with a negative workup for infections and malignancy. She also has persistent MRD positivity indicating molecular relapse. A plan was made to shift to another third-generation TKI, Bosutinib, in January 2022, which has less propensity to cause pleural effusion, which she continues to take to date. She achieved complete hematological remission along with molecular remission while being on Bosutinib, hence bosutinib maintenance was continued. However, 3 months after initiation of Bosutinib, she developed worsening shortness of breath, and a CT scan showed worsening pleural effusion warranting thoracocentesis (Figure 1E). About 850 mL of pleural fluid was removed and the workup revealed exudative pleural effusion with negative cytology. A transthoracic echocardiogram performed during all the hospitalizations did not reveal any cardiac abnormality, and brain natriuretic peptide levels were normal. She was diagnosed with Bosutinib-induced pleural effusion. Shared decision making was made with the patient and family on whether to continue TKI therapy given she achieved molecular remission. A consensus decision was made to continue Bosutinib therapy while pleural effusion management would be with diuretics and thoracocentesis as needed. From April 2022 till date, she requires thoracocentesis at a frequency of once every 3–4 months (Table 1).

In September 2024, she was hospitalized again for worsening shortness of breath, and a CT scan was performed, which showed worsening pleural effusion requiring therapeutic thoracocentesis. Pleural fluid analysis showed exudative effusion and negative workup for infections and malignancy. However, the CT scan this time unlike previous imaging also showed bilateral interstitial opacities, so the possibility of atypical pneumonia was considered and treated with a course of antibiotics (Figure 1F). She responded to the treatment and was discharged in a stable condition. In October 2024, she was readmitted with worsening shortness of breath. A repeat CAT scan showed worsening pneumonia. She was treated with antibiotics. However, her respiratory failure progressively worsened and the family opted for comfort measures only, following which all her drugs including Bosutinib were discontinued. Subsequently, the patient succumbed to her illness.

Naranjo’s protocol for establishing the TKI as the cause of pleural effusion is detailed in Table 2.

## 3. Discussion

Imatinib, a first-generation TKI targeting BCR ABL protein was developed for CML. It acts by binding to the catalytic site of the BCR ABL in its inactive form thereby inhibiting cell proliferation and promoting apoptosis of Bcr-Abl cells [6]. However, mutations in the *Abl* binding site resulted in the development of Imatinib resistance. About 80 specific types of mutations have been observed, of which, the T315I mutation is the most resistant. It warranted developing later-generation TKIs like Dasatinib, Nilotinib, Bosutinib, and Ponatinib. Nilotinib and Ponatinib also block the inactive form of the enzyme like Imatinib, whereas Dasatinib and Bosutinib inhibit the active form of the enzyme [7]. The higher potency of the later-generation TKIs makes these cells susceptible to these drugs despite having the same site of action. Ponatinib is the only effective drug in the presence of T315I mutation [8,9].

Despite having site-specific action, these TKIs also have several other off-target effects depending on the dosage and type of drug. The spectrum of the off-target inhibition is minimal with Nilotinib and maximal with Dasatinib. For example, Imatinib also blocks the platelet-derived growth factor (PDGF) receptors and another stem cell growth factor, Kit; Dasatinib inhibits the proto-oncogene called tyrosine-protein kinase, Src, whereas Ponatinib inhibits the vascular endothelial growth factor receptor [10]. These off-target effects might have a bearing on the clinical outcomes, both in terms of efficacy and side effect profiles, and the prescribing clinicians must be aware of the same. The multitude of target sites by these agents sometimes is used clinically for treating other diseases. As an example, TKIs with EGFR inhibition action are used to treat non-small cell lung cancer [11]. Also, knowing these off-target side effects will help understand the underlying mechanisms of toxicities like pleural effusion, interstitial lung disease (ILD), or pneumonitis [12].

**Figure 2:**
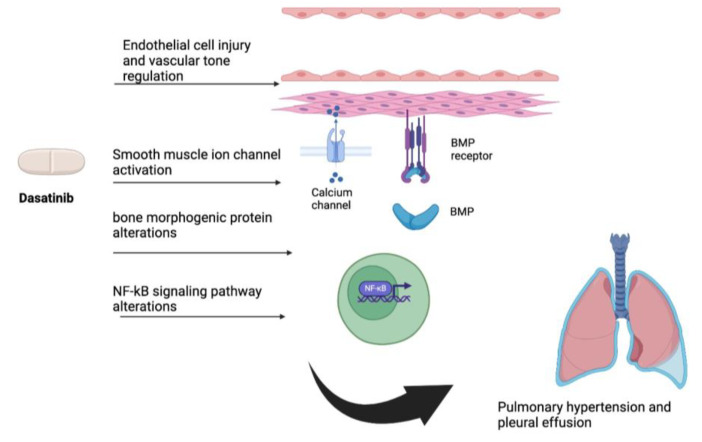
Dasatinib (prototype TKI)-induced pulmonary toxicity. NF-kb: Nuclear factor k-beta; BMP: Bone morphogenic protein.

Pleural effusion is the most common respiratory side effect that may complicate the administration of TKIs. The incidence varies according to the type of TKI, being most common with Dasatinib. Pleural effusion and pulmonary arterial hypertension are two pulmonary side effects observed with Dasatinib use [13]. Although granular details of the cellular events and specific signaling pathways are lacking, these side effects are often reversible after drug withdrawal. Endothelial cell injury, smooth muscle ion channel regulation of vascular tone, alterations in bone morphogenic protein, and alterations in nuclear factor k-beta (NF-KB) signaling pathways are some of the mechanisms described in TKI-related pulmonary toxicities [13,14,15] (Figure 2).

Symptomatic management with diuretics, dose reduction, drug switching, and, finally, drug discontinuation is recommended for managing these complications. For minimal symptoms, these agents can be continued with close monitoring. However, in the case of moderate-to-severe symptoms, drug switching may be attempted as was performed in our patient. Patients who developed pleural effusion while on Dasatinib and who were subsequently treated with Bosutinib may also develop the same side effect as seen in our patient, though rare [16,17]. In a cohort study including 20 patients developing pleural effusion while on Dasatinib and who subsequently received Bosutinib, 30% of them had experienced a recurrence [18]. 

Bosutinib-associated pleural effusion is rare and was seen in less than 5% of patients during a follow-up period of 5 years. Older adults are more susceptible to this complication for some unknown reason [17]. One case of Bosutinib-related concomitant pneumonitis along with pleural effusion has also been reported in the literature [19]. Chylothorax has also been reported with Bosutinib usage. Our case adds to the minimal available literature (Table 3). Ponatinib is a third-generation TKI that acts and blocks the inactive form of the enzyme. No cases of pleural effusions or interstitial pneumonitis have been specifically associated with ponatinib to date, apart from one case of interstitial lung disease reported in the phase 1 study evaluating the effect of ponatinib in refractory CML [9].

## 4. Conclusions

Through this case report, we would like to highlight refractory recurrent pleural effusion caused by Bosutinib, adding to the lacking available literature. In addition, we highlight the various treatment options in patients having cross-intolerance to various TKIs, especially pulmonary toxicity, and Ponatinib might be a suitable option in such cases.

## Figures and Tables

**Table 1:** TKI time frame and reason for discontinuation.

Time Frame	TKI Type	Drug Dosage	Disease Status	Reason for Discontinuation
2015 to 17 February 2016	Imatinib	400 Mg BID	CHR, MMRD positive	Drug-induced TCP
March 2016–April 2016	Imatinib	400 Mg BID	CHR, MMRD positive	Possibility of drug induced TCP and MRD relapse
April 2016–March 2019	Dasatinib	100 Mg Daily	CHR, MMRD positive (p190 transcripts detected at level of %BCR/ABL1: ABL1: 0.0635)	Pleural effusions and molecular relapse.
March 2019–January 2022	Nilotinib	400 Mg BID	CHR, MMRD positive	Recurrent pleural effusions and Coronary artery calcifications. And Molecular relapse.
March 2022 to Currently Active	Bosutinib	400 Mg Daily, later shifted to 200 Mg DAILY	CHR, MMRD positive	Currently Active

TKI time frame and reason for discontinuation in the index patient. BID: twice daily; MG: milligrams; CHR: Complete Hematological remission; MMRD: Molecular measurable residual disease; TCP: Thrombocytopenia; MRD: Measurable residual disease. MMRD is measured by quantitative polymerase chain reaction (q PCR). The same method has been employed every time while measuring MMRD. This assay only detects BCR/ABL1 p210 (b2a2 and b3a2; major breakpoint region) and p190 (e1/a2) fusion transcripts. Other BCR/ABL1 fusion transcripts including p230 (e19/a2) will not be detected. Methodology: ABL1 and BCR/ABL1 transcript levels for p210 (b2a2 and b3a2; major breakpoint region) and/or p190 (e1a2; minor breakpoint region) are determined by performing quantitative, reverse transcription PCR of extracted RNA. The assays’ detection limit is approximately 0.002%IS or MR 4.7 for p210 and 0.0025% BCR/ABL1:ABL1 for p190. Detection with this assay is an MRD-positive disease.

**Table 2:** Naranjo’s Algorithm for the probability of establishing ADR in the index patient [5].

Questionnaire	+1/+2	0	−1
Are there previous conclusive reports on this reaction?	+1	-	**-**
Did the adverse events appear after the suspected drug was given?	+2	-	**-**
Did the adverse reaction improve when the drug was discontinued, or a specific antagonist was given?	+1	-	**-**
Did the adverse reaction appear when the drug was re administered?	+2	-	**-**
Are there alternative causes that could have caused the reaction?	+1	-	**-**
Did the reaction reappear when a placebo was given?	-	0	**-**
Was the drug detected in any body fluid in toxic concentrations?	-	0	**-**
Was the reaction more severe when the dose was increased, or less severe when the dose was decreased?	+1	-	**-**
Did the patient have a similar reaction to the same or similar drugs in any previous exposure?	+1	-	**-**
Was the adverse event confirmed by any objective evidence?	+1	-	**-**

Naranjo’s Algorithm for the probability of establishing ADR in the index patient: >/= 9 indicating a definite Adverse Drug Reaction. (1) There are previous reports of Bosutinib-induced pleural effusion making it +1; (2) adverse events appeared after TKI was given making it +2; (3) yes there was an improvement in pleural effusion once the dose was reduced as noticed in the patient’s clinical course making it +1; (4) yes, the adverse reaction did appear after the drug dose was increased making it +2; the dose–response relationship (+1); similar adverse effects with all other class of drugs (+1); and pleural fluid analysis being exudative ruling out other causes makes TKI-related pleural effusion a likely diagnosis (+1).

**Table 3:** Literature on Bosutinib induced pleural effusion.

Author and Year	Age/Gender	Primary Diagnosis	TKI Related Pleural Effusion	Management
Aslan et al., 2023 [20]	59/M	CML	Nilotinib, Dasatinib, and Bosutinib	Steroids and Shifted to Ponatinib
Moguillansky et al., 2017 [21]	71/M	CML	Bosutinib	Bosutinib discontinued, Steroids, Diuretics, and underwent Allo HSCT
Index case	74/F	Ph + ALL	Dasatinib, Nilotinib, and Bosutinib	Diuretics and Thoracocentesis.

## Data Availability

Data are contained within the article.
